# The Promotoer, a brain-computer interface-assisted intervention to promote upper limb functional motor recovery after stroke: a study protocol for a randomized controlled trial to test early and long-term efficacy and to identify determinants of response

**DOI:** 10.1186/s12883-020-01826-w

**Published:** 2020-06-27

**Authors:** Donatella Mattia, Floriana Pichiorri, Emma Colamarino, Marcella Masciullo, Giovanni Morone, Jlenia Toppi, Iolanda Pisotta, Federica Tamburella, Matteo Lorusso, Stefano Paolucci, Maria Puopolo, Febo Cincotti, Marco Molinari

**Affiliations:** 1 Fondazione Santa Lucia, IRCCS, Rome, Italy; 2 Department of Computer, Control, and Management Engineering “Antonio Ruberti”, Sapienza University of Rome, Rome, Italy; 3 Istituto Superiore di Sanità, Rome, Italy

**Keywords:** EEG-based brain-computer interface, Stroke, Hand functional motor recovery, Brain plasticity, Motor learning, Motor imagery, Neurorehabilitation

## Abstract

**Background:**

Stroke is a leading cause of long-term disability. Cost-effective post-stroke rehabilitation programs for upper limb are critically needed. Brain-Computer Interfaces (BCIs) which enable the modulation of Electroencephalography (EEG) sensorimotor rhythms are promising tools to promote post-stroke recovery of upper limb motor function. The “Promotoer” study intends to boost the application of the EEG-based BCIs in clinical practice providing evidence for a short/long-term efficacy in enhancing post-stroke hand functional motor recovery and quantifiable indices of the participants response to a BCI-based intervention. To these aims, a longitudinal study will be performed in which subacute stroke participants will undergo a hand motor imagery (MI) training assisted by the Promotoer system, an EEG-based BCI system fully compliant with rehabilitation requirements.

**Methods:**

This longitudinal 2-arm randomized controlled superiority trial will include 48 first ever, unilateral, subacute stroke participants, randomly assigned to 2 intervention groups: the BCI-assisted hand MI training and a hand MI training not supported by BCI. Both interventions are delivered (3 weekly session; 6 weeks) as add-on regimen to standard intensive rehabilitation. A multidimensional assessment will be performed at: randomization/pre-intervention, 48 h post-intervention, and at 1, 3 and 6 month/s after end of intervention. Primary outcome measure is the Fugl-Meyer Assessment (FMA, upper extremity) at 48 h post-intervention. Secondary outcome measures include: the upper extremity FMA at follow-up, the Modified Ashworth Scale, the Numeric Rating Scale for pain, the Action Research Arm Test, the National Institute of Health Stroke Scale, the Manual Muscle Test, all collected at the different timepoints as well as neurophysiological and neuroimaging measures.

**Discussion:**

We expect the BCI-based rewarding of hand MI practice to promote long-lasting retention of the early induced improvement in hand motor outcome and also, this clinical improvement to be sustained by a long-lasting neuroplasticity changes harnessed by the BCI-based intervention. Furthermore, the longitudinal multidimensional assessment will address the selection of those stroke participants who best benefit of a BCI-assisted therapy, consistently advancing the transfer of BCIs to a best clinical practice.

**Trial registration:**

Name of registry: BCI-assisted MI Intervention in Subacute Stroke (Promotoer).

Trial registration number: NCT04353297; registration date on the ClinicalTrial.gov platform: April, 15/2020.

## Background

Stroke is a major public health and social care concern worldwide [1]. The upper limb motor impairment commonly persists after stroke, and it represents the major contribution to long-term disability [2]. It has been estimated that the main clinical predictor of whether a patient would come back to work is the degree of upper extremity function [3]. Despite the intensive rehabilitation, the variability in the nature and extent of upper limb recovery remains a crucial factor affecting rehabilitation outcomes [4].

Electroencephalography (EEG)-based Brain-Computer Interface (BCI) is an emerging technology that enables a direct translation of brain activity into motor action [5]. Recently, EEG-based BCIs have been recognized as potential tools to promote functional motor recovery of upper limbs after stroke (for review see [6]). Several randomized controlled trials have shown that stroke patients can learn to modulate their EEG sensorimotor rhythms [7] to control external devices and this practice might facilitate neurological recovery both in subacute and chronic stroke phase [8–10].

We were previously successful in the design and validation of an EEG sensorimotor rhythms–based BCI combined with realistic visual feedback of upper limb to support hand motor imagery (MI) practice in stroke patients [11, 12]. Our previous pilot randomized controlled study [8] with the participation of 28 subacute stroke patients with severe motor deficit, suggested that 1 month BCI-assisted MI practice as an add-on intervention to the usual rehabilitation care was superior with respect to the add-on, 1 month MI training alone (ie., without BCI support) in improving hand functional motor outcomes (indicated by the significantly higher mean score at upper extremity Fugl-Meyer scale in the BCI with respect to control group). A greater involvement of the ipsilesional hemisphere, as reflected by a stronger motor-related EEG oscillatory activity and connectivity in response to MI of the paralyzed trained hand was also observed only in the BCI-assisted MI training condition. These promising findings corroborated the idea that a relatively low-cost technique (i.e. EEG-based BCI) can be exploited to deliver an efficacious rehabilitative intervention such as MI training and prompted us to undertake a translational effort by implementing an all-in-one BCI-supported MI training station– the Promotoer [13].

Yet, important questions remain to be addressed in order to improve the clinical viability of BCIs such as defining whether the expected early improvements in functional motor outcomes induced by the BCI-assisted MI training in subacute stroke [8] can be sustained in a long-term as it has been shown for other BCI-based approaches in chronic stroke patients [10, 14]. This requires advancements in the knowledge on brain functional re-organization early after stroke and on how this re-organization would correlate with the functional motor outcome (evidence-base medicine). Last but not least, the definition of the determinants of the patients response to treatment is paramount to optimize the process of personalized medicine in rehabilitation. We will address these questions by carrying out a randomized trial to eventually establish the fundamentals for a cost-effective use of EEG-based BCI technology to deliver a rehabilitative intervention such as the MI in hospitalized stroke patients.

### Aim and hypotheses

The “Promotoer” study is a randomized controlled trial (RCT) designed to provide evidence for a significant early improvement of hand motor function induced by the BCI-assisted MI training operated via the Promotoer and for a persistency (up to 6 months) of such improvement. Task-specific training was reported to induce long-term improvements in arm motor function after stroke [15–17]. Thus, our hypothesis is that the BCI-based rewarding of hand MI tasks would promote long-lasting retention of early induced positive effect on motor performance with respect to MI tasks practiced in an open loop condition (ie, without BCI). Accordingly, the primary aim of the “Promotoer” RCT will be first to determine whether the BCI based intervention (MI-BCI) administered by means of a BCI system fully compatible with a clinical setting (the Promotoer), is superior to a non-BCI assisted MI training (MI Control) in improving hand motor function outcomes in sub-acute stroke patients admitted to the hospital for their standard rehabilitation care; secondly, we will test whether the efficacy of BCI-based intervention on hand motor function outcomes is sustained long-term after the end of intervention (6 months follow-up). A further hypothesis is that such clinical improvement would be sustained by a long-lasting neuroplasticity changes as harnessed by the BCI–based intervention. This hypothesis rises from current evidence for an early enhancement of post-stroke plastic changes enabled by BCI-based trainings [8–10]. To test this hypothesis, a longitudinal assessment of the brain network organization derived from advanced EEG signal processing (secondary objective) will be performed.

The heterogeneity of stroke makes prediction of treatment responders a great challenge [18]. The potential value of a combination of neurophysiological and neuroimaging biomarkers with the clinical assessment in predicting post-stroke motor recovery has been recently highlighted [19]. Our hypothesis is that the longitudinal combined functional, neurophysiological and neuroimaging assessment over 6 months from the intervention will allow for insights into biomarkers and potential predictors of patients response to the BCI-Promotoer training (secondary aim). To this purpose, well-recognized factors contributing to recovery after stroke such as the relation between clinical profile, lesion characteristics and patterns of post-stroke motor cortical re-organization (eg., ipsilesional/contralesional primary and non-primary motor areas, cortico-spinal tract integrity, severity of motor deficits at baseline; for review see [19]) will be taken into account.

## Methods/design

We will test our hypotheses by randomizing 48 participants in a single center, single blind, RCT to investigate the early and long-term effectiveness of a BCI-assisted MI training for hand movements delivered during the subacute phase of stroke (hospitalization period) and at several follow-up time points (outpatient period). The primary and secondary outcome measures collected from participants randomized to BCI-assisted MI training will be compared to those of participants administered an equivalent dose of MI training without the support of BCI (MI Control group).

All the procedure conducted in the present trial are in compliance with the national institutional ethical standards and with the Helsinki Declaration. The study protocol and related procedures are approved by the institutional review board: the Independent Ethical Committee of the Fondazione Santa Lucia (FSL), IRCCS, Rome, Italy (protocol # CE/PROG.755 11–06-19).

### Study design

**Fig. 1:**
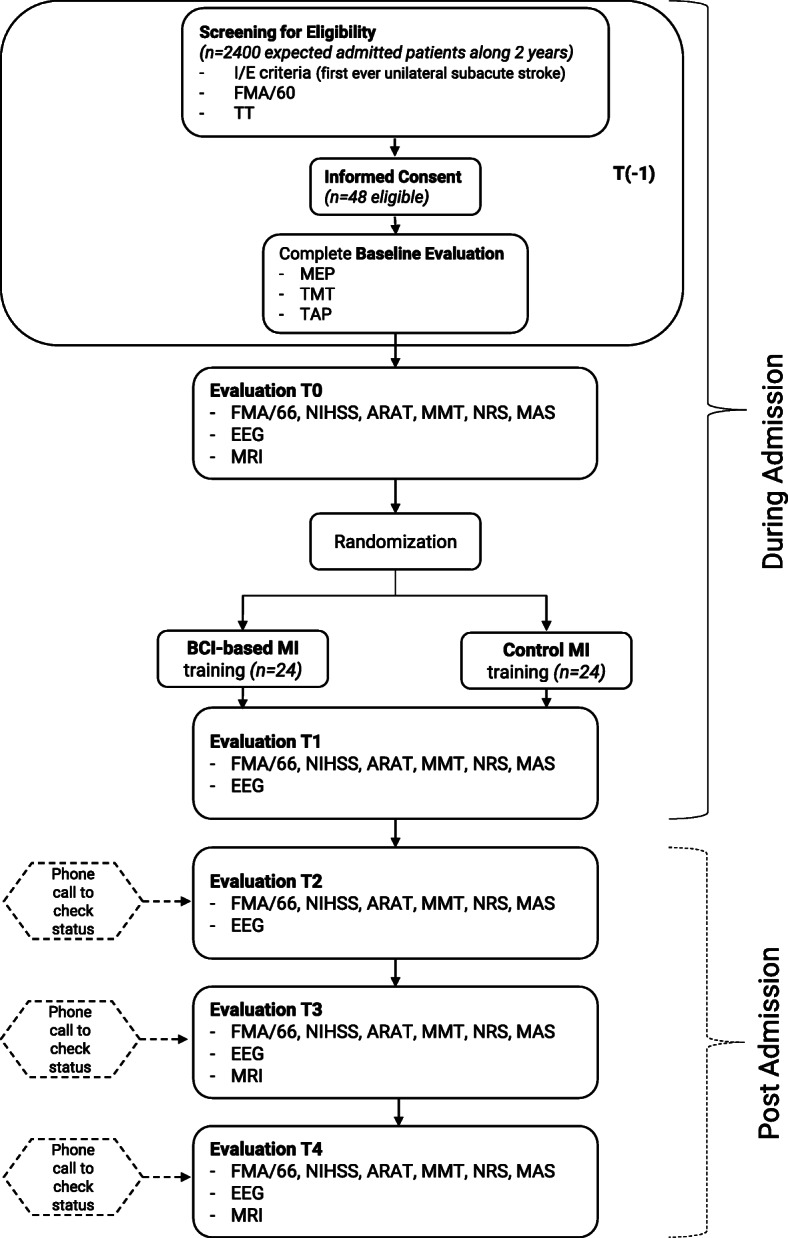
Promotoer trial summary. All admitted participants (approximately 2400 along the course of two years) will be screened (T(− 1)) for eligibility according to I/E (Inclusion/Exclusion) criteria. The FMA/60 (Fugl-Meyer Assessment, upper limb section, without the 6 points relative to the reflexes [20];) and the TT (Token Test) to verify the participants ability to understand the task, will be used for Eligibility and Stratification. Eligible participants will be presented with the Informed Consent and recruited (48 participants). After recruitment, screening evaluation (T(− 1)) will be completed with the execution of MEP (Motor Evoked Potentials, upper limbs) and other neuropsychological tests (TMT - Trial Making Test; TAP - Test of Attentional Performance). Clinical functional outcome variables will be assessed at T0, T1, T2, T3 and T4. EEG (electroencephalogram) will also be performed at T0, T1, T2, T3 and T4. MRI (Magnetic Resonance Imaging) scan of the whole brain will be performed at T0, T3 and T4. Enrolment, Randomization, Training and Evaluations up to T1 will be performed while participants are admitted for rehabilitation. Subsequent evaluations (T2, T3, T4) will most likely occur at discharge (post-admission) and will be preceded by a phone call to check participants status and to set appointments

The Promotoer trial is designed as a randomized, controlled, assessors blinded single-center superiority trial with 2 parallel groups with 1:1 allocation ratio. The participants recruitment, intervention delivery and data collection will take place at Fondazione Santa Lucia, IRCCS, Rome Italy. Upon admission (no later than 48–72 h) to the FSL Hospital for post-stroke standard neurorehabilitation care, subacute stroke participants will be screened for eligibility criteria by the project clinical staff (neurologists, neuropsychologists, neurophysiologists). Prospective participants are first identified through a review of admissions to the inpatient rehabilitation wards that is carry out by staff neurologists. Screening for eligibility (T(− 1)) includes clinical/functional, and neuropsychological measurements (see below, Inclusion criteria section and Table 1). Data will be recorded in an ad hoc Case Report Form (CRF; details in Data collection and management section). The eligible participants will be introduced to the study protocol by authorized personnel and they will be offered the informed consent. Once the Study Informed Consent is signed and the enrollment is effective, baseline evaluation is completed and participants are randomized (within one week from the first contact with the participants) to one of the 2 intervention groups (as add-on): the BCI-assisted MI training (experimental group, BCI-MI) or the MI training with no BCI (control group Control-MI;). The primary outcome is the Fugl-Meyer Assessment (FMA) score for upper extremity (UE-FMA) collected at T1 (end of treatment). Secondary outcomes are the UE-FMA score in the long-term (up to 6 months after the end of intervention), the Minimal Clinically Important Difference (MCID) improvement of 7 points at UE-FMA and a battery of clinical/functional scales to monitor upper limb muscle strength, tone, pain, and functional ability. Other pre-specified outcome measures are extracted from neuropsychological, neurophysiological and neuroimaging assessment at different timepoints. The overall outcome assessments are detailed in Assessment section. Outcome assessors are blinded to treatments. The study flow is illustrated in Fig. 1.

**Table 1:**
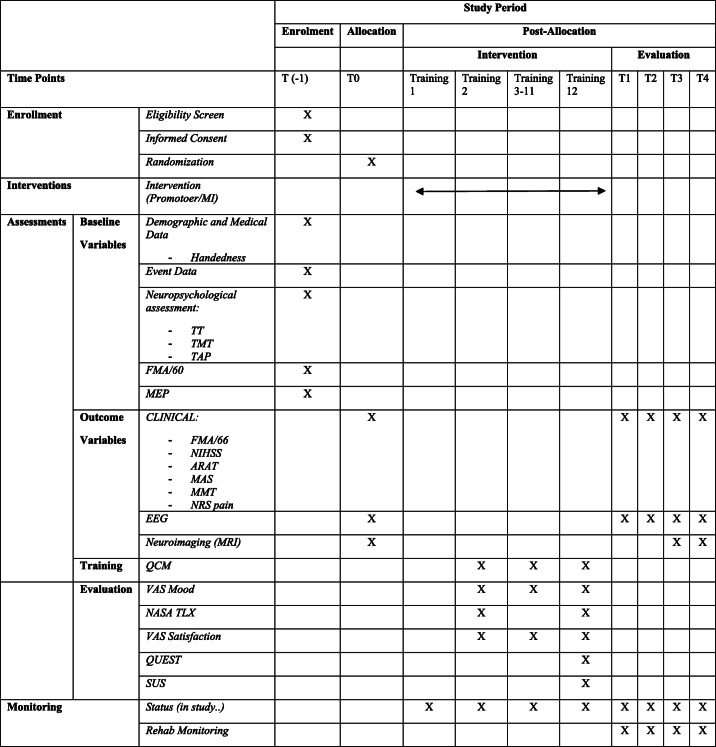
STANDARD PROTOCOL ITEMs as recommended for Interventional Trials (SPIRIT)

The study is presented according to the Standard Protocol Items: Recommendations for Interventional Trials (SPIRIT) [21]. Table 1 shows the SPIRIT schedule of enrolment, interventions and assessments.

### Inclusion/exclusion criteria

Participants are adults admitted to FSL Hospital with a diagnosis of subacute, first ever, unilateral cerebral stroke (ischemic or intraparenchymal hemorrhagic stroke). Magnetic Resonance Imaging (MRI) will be used to confirm the clinical and history-chart criteria (at baseline) for unilateral stroke. Specific Inclusion and Exclusion criteria are listed below:

Inclusion criteria:

- age between 18 and 80 years;
- Current admission at FSL site for rehabilitation care;
- first ever unilateral stroke, confirmed by MRI;
- hemiplegia/hemiparesis from 1 to 6 months since stroke; for eligibility and stratification we will employ UE-FMA. According to [20], we will include severe and moderate stroke participants as defined by the UE-FMA scoring without the 6 points relative to reflexes. Therefore, severe participants with scores from 0 to 19 (out of a maximum of 60 scores) and moderate participants with scores from 20 to 47 (out of 60 scores) will be included.

Exclusion criteria:

- severe neglect and aphasia;
- dementia;
- severe spasticity– Modified Ashworth Scale (MAS) > 4 at shoulder/elbow/wrist;
- UE-FMA > 47 (out of 60 score, see above) will define mild participants who will be excluded from the current study;
- Token Test (TT) < 29 score: TT [22] is commonly used to detect receptive language disorders in aphasic participants, who do not show comprehension disorders in normal communicative interaction; participants with a score value lower than the cut-off value of 29 score will be excluded from the study;
- concomitant neurological disorders;
- concomitant enrollment in other clinical research trials which are based on neuromodulation (such as repetitive Transcranial Magnetic Stimulation (TMS), transcranial direct current stimulation), robotics, virtual reality and cognitive training requiring mental imagery even if the content of imagination is different from that of the study intervention.

### Assessments

#### Outcome assessments

The outcome measures refer to as all assessments which will be performed both pre and post- intervention and at follow-up. The different evaluation timepoints are: T0 = at randomization, before intervention; T1 = at end of intervention training (within 48 h; primary time point); T2 = at 1 month from end of training; T3 and T4 = at 3 and 6 months from end of training, respectively (see SPIRIT diagram in Table 1 for details).

Trained clinical/research staff neurologists, neuropsychologists, neurophysiologists, therapists and an expert neuroanatomy researcher will perform the assessments and will be blind to participant intervention allocation with the exception of recruiting neurologists who are not blinded (see Study Design section). An ad hoc CRF will be used to record all type and timing assessments (details in Data collection and management section).

#### Primary

The UE-FMA (motor and sensory part) score (mean ± standard deviation, SD; maximum score 66 equal to normal) at T1 (end of treatment) is the primary outcome measure for the trial and the basis for the sample size calculation. The UE-FMA is widely recommended for the evaluation of sensorimotor impairments in stroke rehabilitation research [23, 24].

#### Secondary

The secondary outcome measures are the UE-FMA score (mean ± SD) at T2, T3, and T4; the MCID [25] improvement sets at 7 points at UE-FMA at T1 and follow-up (T2, T3, T4); the MAS for Spasticity [26] score (mean ± SD) for shoulder/elbow/wrist at T1 T2, T3 and T4; the Numeric Rating Scale (NRS) for pain (affected upper limb; 0 = no pain; 1… 9; 10 unspeakable pain) at T1, T2, T3 and T4; the Action Research Arm Test for upper limb function (ARAT [27]) score (mean ± SD) at T1, T2, T3 and T4; the National Institute of Health Stroke Scale (NIHSS; min 0-max 4 depending on items; 0 = normal [28];) score (total score, mean ± SD) at T1, T2, T3 and T4; the Manual Muscle Test (MMT; min 0 max 5; 0 = no movements; total score, mean ± SD) for shoulder/elbow/wrist (flexor/extensor muscles) at T1, T2, T3 and T4.

Other pre-specified measures will be extracted from neurophysiological, neuroimaging and neuropsychological assessments at different timepoints in order to evaluate: i) neurophysiological correlates of the BCI-MI training functional efficacy; ii) possible factors influencing the response to experimental intervention (BCI-MI training); and iii) the usability/acceptability of the technology-based training. These assessments are:

#### Neurophysiological assessment

This includes:

- the Motor Evoked Potentials (MEPs) to evaluate the corticospinal tract integrity at screening (T(− 1)); MEPs will be recorded from the bilateral Abductor Digiti Minimi (ADM) muscle by following the methodology reported in [29]. In brief, TMS will be delivered in single stimuli at maximum output stimulator (5 stimuli for each side) and electromyographical (EMG) activity will be recorded from ADM muscle bilaterally, if allowed during voluntary contraction. EMG traces will be superimposed to determine MEP latency. Peripheral motor conduction time (PMCT) will be obtained by recording Compound Muscle Action Potentials (CMAP) and F-waves from ulnar nerves bilaterally. PMCT will be calculated as (CMAP latency + F wave latency – 1)/2. Central motor conduction time will be calculated as MEP latency – PMCT.
- The high-density EEG recordings (motor relevant oscillatory activity and functional connectivity estimation) to evaluate the short and long-term neurophysiological substrates of the experimental intervention efficacy at T0, T1, T2, T3 and T4 (ie., to monitor plasticity phenomena possibly correlated with functional recovery) and to identify offline the BCI control features for each participant (see Intervention - *BCI-assisted motor imagery training (experimental intervention)* section). All participants will be comfortably seated in an armchair (or directly on their wheelchair) in a dimly lit room (laboratory) with their upper limbs resting on a pillow. EEG data will be collected from 61 positions, assembled on an active electrode cap (g.GAMMAcap equipped with g.SCARABEO active electrodes, g.tec medical engineering GmbH Austria) according to an extension of the 10–20 International System (ground: left mastoid, reference: auricular lobes). EMG data will be collected from the extensor digitorum (ED) and flexor digitorum superficialis (FDS) muscles of the affected and unaffected upper limb. Biosignals will be digitalized at 256 Hz and amplified by the g.HIamp amplifier (g.tec medical engineering GmbH Austria). Four minutes of EEG recording at rest (eyes closed and opened) will be acquired at the beginning of session. Each session will consist of 4 runs, 40 trials each. Each run will consist of 2 tasks: i) a hand kinesthetic MI [30] task which will consist of either hand closing or opening, (identical to those performed during the intervention training) and it will involve either the affected (ie., paralized) and unaffected hand and, ii) rest. Each run will include 20 ± 1 MI and 20 ± 1 rest trials with a randomized order. At the beginning of the session, participants will be allowed to actually execute the imagined motor tasks with unaffected and affected hand (whenever possible) for a limited number of trials in order to facilitate task comprehension and the rehearsal of sensorimotor sensations evoked by the actual execution during MI performance. Instructions for the participant will be “Imagine to close/open your hand in first person by trying to feel the sensations as if you were actually performing the movement”. Visual cues will be presented as visual representation of two equally illuminated hands on a black screen for 3 s, then the hand designated for the specific run (unaffected or affected) will lit-up. Participants will be instructed to imagine the hand movement for 4 s, then both hands will disappear and the trial ends. In rest trials, both hands will be presented to the participants for the whole trial duration (7 s) after which a black screen will appear. Each trial will be also defined by a concomitant written instruction on the screen (“rest” “image hand closing” “image hand opening”). During intertrial interval, participants will be confronted with a black screen. The total trial duration will be 8.5 s with an intertrial interval of 1.5 s. Changes of the motor relevant oscillatory activity will be measures as significant changes in the Power Spectral Density (PSD) maps (details in [8]). The PSD analysis will be performed offline on the high-density EEG data recorded at T0, T1, T2, T3 and T4, to describe the differences between the BCI-assisted MI and MI control groups. After pre-processing and processing of the EEG signals, the PSD values will be computed and averaged within the EEG frequency bands of interest (theta, alpha, beta1, beta2 and gamma). Statistical PSD maps will be generated for each participant dataset by contrasting MI and rest PSD values for each channel and frequency band for MI tasks performed with the affected and unaffected hand. Significant between-group (BCI-MI and Control-MI) differences in the PSD maps from each participant (considering each participant as “repetition”) at different timepoints will be also evaluated. Changes of the brain networks at different timepoints will be measured as significant changes in the EEG-derived functional connectivity under rest (resting state) and task conditions (MI with affected and unaffected hand). We will adopt the Partial Directed Coherence (PDC) estimator that is a well-established, full multivariate spectral measure that determines the directed influences between a pair of signals in a multivariate dataset [31]. The PDC matrices will be validated applying asymptotic statistical procedure that allows for discarding the spurious links due to random correlation between the data, returning a significance threshold for each PDC value [32]. The PDC matrices (expressed as weighted directed adjacency matrices) will be computed for EEG data recorded at different timepoints from both BCI-assisted MI and control MI groups and for the EEG frequency band of interest. A graph theory-based approach will be adopted to provide synthetic measures that described the topological properties of the brain network [33] both at single-subject and group level. Advanced analyses of the EEG/EMG data will be under the supervision of expert partner Sapienza University, Rome Italy (SAP).

#### Neuroimaging assessment

This includes structural MRI of the whole brain to evaluate lesion size and white matter alteration as factors influencing the intervention response at T0, T3 and T4. The MRIs will be collected with Siemens 3.0 T Prisma scanner at FSL Neuroimaging Unit. Structural scans are performed with T1-weighted MP-RAGE (1.0 mm isotropic voxels; TE = 226 ms,TR = 1950 ms, flip angle = 9 °), a transverse T2-weighted turbo spin echo sequence, (1.0 mm isotropic voxels; TE = 442 ms, TR = 2500 ms) the T2W images are acquired to localize the lesions. Routine T2Wand DW images are acquired to specify better the type and size lesions. Each MRI volume are transformed into the Montreal Neurological Institute Space (2012) by means of Register (http://www.bic.mni.mcgill.ca/ServicesSoftwareVisualization/Register). Voxel based morphometry analysis will be performed to estimate lesion volume. The Corpus Callosum volume will be also analyzed in order to evaluate the role of interhemispheric connections integrity in the response to treatment of participants.

#### Neuropsychological and user experience assessment

The neuropsychological assessment includes the Test for Attentional Performance [34] (3 items: alertness, sustained attention and working memory) and the Trail Making Test [35] performed at screening (T(− 1)).

Finally, the User experience assessment of the technology-based training will include:

- the Questionnaire on Current Motivation [12, 36] (4 items) performed at each single training session (starting from session 2) to evaluate motivation and adherence to technology;
- the Workload NASA-TLX [37] Total score to evaluate mental workload-fatigue during training with technology at second and last training session;
- the System Usability Score (SUS) [38] to evaluate usability of technology at T1;
- the Quebec User Evaluation of Satisfaction with assistive Technology 2.0 (QUEST 2.0) [39] at the T1 to evaluate satisfaction with technology.

### Randomization procedure and methods

The random allocation sequence of participants to experimental (BCI-MI training) or control (Control-MI training) intervention groups will be generated by using the Ralloc procedure of the Statistical software STATA (https://www.stata.com/). The randomization sequence will be stratified by side of stroke lesion (left/right hemisphere) and by the baseline score of UE-FMA such as severe (UE-FMA score of < 19/60 with 60 as maximum score) versus moderate (UE-FMA score from 20 to 47/60 with 60 as maximum score) [20]. A 1:1 allocation ratio, and permuted blocks of varying size will be used. For each stratum, the sequence will include 48 random allocations. Randomization assignment is obtained from the trial statistician at Istituto Superiore Sanità (ISS, Rome Italy). Sealed envelopes are transferred to study protocol staff clinicians (recruiting neurologists) at FSL Hospital and are securely stored. The allocation sequence is also stored at ISS. Randomization of participants will stop after randomization of the 48th participant in the study by summing participants in all strata.

### Intervention

#### BCI-assisted motor imagery training (experimental intervention*)*

**Fig. 2:**
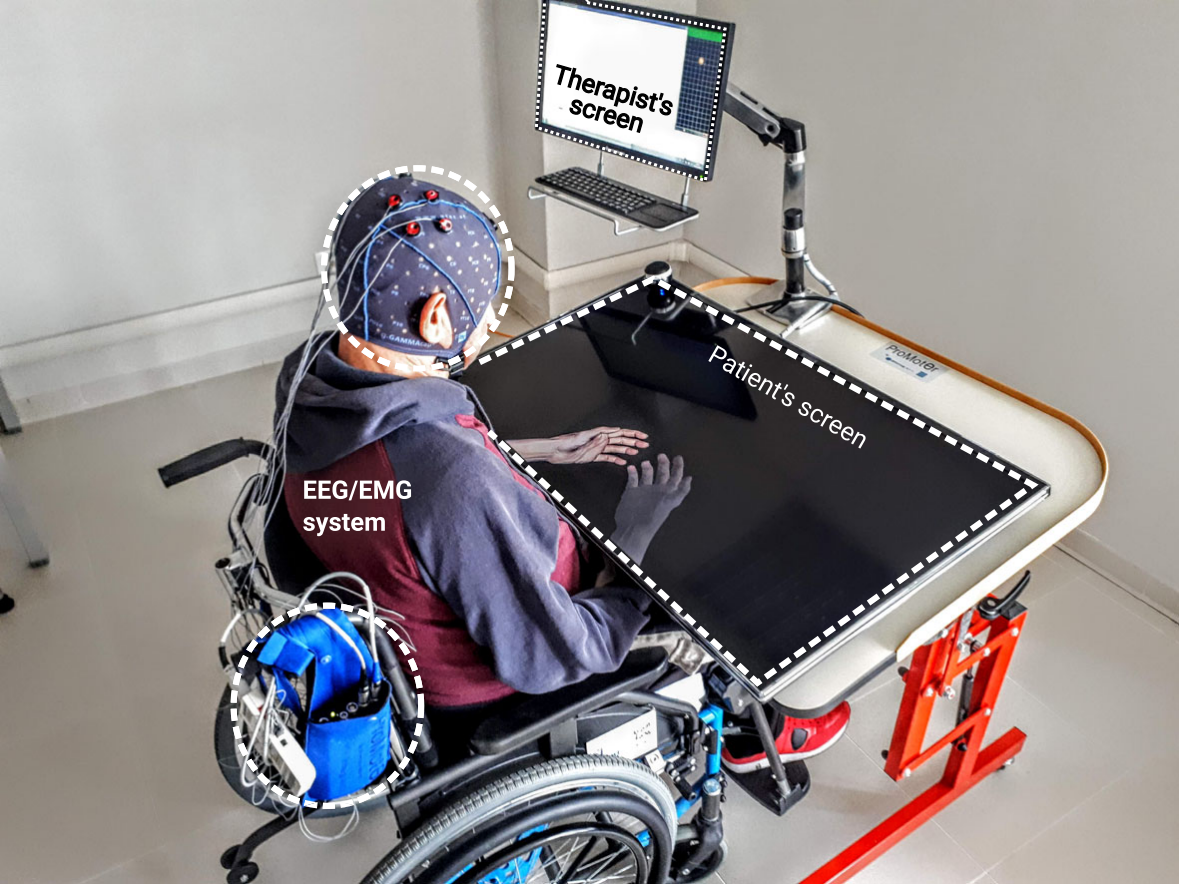
The Promotoer system. The Promoter is equipped with a computer, a commercial wireless EEG/EMG system (g.MOBIlab, g.tec medical engineering GmbH Austria), a screen for the therapist feedback (for the electroencephalographic - EEG activity and electromyographic- EMG activity monitoring) and screen for the ecological feedback to the participant; this ecological feedback is delivered by means of a custom software program that provides for (personalized) visual representation of the participant’s own hands. As such, this software allows the therapists to create an artificial reproduction of a given participant’s hand and forearm by adjusting a digitally created image in shape, size, skin color and orientation to match as much as possible the real hand and arm of the participant. Real-time feedback is provided by means of BCI2000 software [40]. The degree of EEG desynchronization over selected electrodes within selected frequencies (BCI control features) determines the vertical velocity of the cursor on the therapist’s screen and it operates the “virtual” hand software accordingly. The image is original as it is owned by the authors

The system Promotoer is an all-in-one BCI-supported MI training station which allows users to practice the kinesthetic MI [30] of hand movements such as closing and opening in a close-loop condition (details are illustrated in Fig. 2).

The BCI control features, namely those relevant, significant frequency/spatial changes of the EEG signals that are to drive the visual feedback to therapist (cursor motion on therapist screen in Fig. 2) and to operate the “virtual” hand software (feedback to participants in Fig. 2) will be extracted through offline analysis of the MI-related EEG data from the high-density EEG recording at T0 (baseline session; see Neurophysiology Assessment section). A custom semiautomatic, physiologically-driven EEG feature selection method implemented in MATLAB environment (The MathWorks, Inc., Natick, Massachusetts, USA) as a user-friendly interface GUIDER [41] will be used to process EEG data. In brief, the GUIDER interface is programmed to spatially filter EEG data by means of spatial bipolar filters [42], to divide EEG data into 1 s-length epochs and to eventually extract all spectral features (ie., spectral amplitude at each frequency bin for each channel) in a reasonable range (0-36 Hz) for each epoch using maximum entropy method [43] (16th order model, 2 Hz resolution, no overlap).

A priori criteria for the selection of the relevant control features are then applied: i) features are derived only from the (fronto-) central and centroparietal electrodes that are distributed over the lesioned hemisphere; ii) they show desynchronization patterns (ie, a decrease in spectral power [7]) at EEG frequencies that are typical for the modulation of sensorimotor rhythms induced by the MI tasks. Thus, through BCI training, we aim to reinforce the individual EEG patterns of reactivity that most resembles the physiological activation that is relevant to movement imagination of the contralateral hand [44]. Qualified neurophysiologists will be instructed to use the GUIDER tool and will flag for each single participant the stroke lesioned hemisphere and EEG channels over the scalp sensorimotor areas to be included in the analysis. GUIDER will then extract (regression modeling) the optimal subset of predictor variables that best discriminate between the dependent variables (eg., task vs rest) and it will return an external parameter file ready to be loaded on BCI2000 software [40] for the training sessions.

#### Motor imagery training without BCI support (control intervention)

Based on our previous findings on the efficacy of a BCI-assisted MI training in subacute stroke participants [8] and according to the primary aim of the present interventional study, the control intervention is an equivalent dose-setting MI training, but without BCI support. The Control-MI intervention training will be implemented under the same conditions as the experimental intervention (BCI-MI training). Specifically, the Promotoer station (Fig. 2) will be used to provide participants only with a visual cue of the MI trial duration that is, the visually represented hand stands still with no BCI control. Participants will perform the same MI tasks (opening/closing hand trial, in random order) as in the experimental intervention training.

#### Training session

During training sessions, all participants will be seated on a comfortable chair in a dimly lit room with their hands and forearms resting under the Promotoer screen (Fig. 2). They will be instructed by the therapists to perform the kinesthetic imagination (MI) only of the affected (i.e. paralyzed) hand movements either closing or opening in separate runs randomly ordered. Each training session will consist of 4 runs (20 trials each). In the case of BCI-MI training, each trial will consist of constant baseline period of 4 s and MI task period of maximally 10s in case of unsuccessful trial, whereas for the Control-MI training the trial length is fixed at 4–6 s (equivalent to a mean successful trial duration as per BCI modality).

In both intervention training, EEG data will be collected from 8 EEG electrodes in bipolar fashion, assembled on an electrode cap according to an extension of the 10–20 International System (ground: left mastoid). The electrode positions will cover the bilateral sensorimotor area with a bipolar montage customized for each participant according to the individual significant EEG features (extracted as described in *BCI-assisted motor imagery training (experimental intervention))* section that only in the case of the BCI-MI training will serve as BCI control features. EMG activity will be continuously recorded through surface electrodes places over the ED and FDS muscles of the affected hand in order to ensure that no actual movement is performed during training (only MI). EEG and EMG data will be digitalized at 256 Hz.

#### Dose of intervention training, usual care and follow-up monitoring

Both the BCI-MI and Control-MI intervention training will comprise a total of 12 sessions with a week delivery of 3 times/week, 45 min duration. Training will be completed in 4–6 weeks. Adherence to treatment is fixed as 9 to 12 intervention training sessions.

The study interventions are conceived as add-on with respect to the standard rehabilitation care (treatment as usual; TAU) hence, all enrolled participants will undergo their standard rehabilitation program as indicated by the clinicians during the hospitalization. To ensure between and within group comparability, the TAU will be delivered according to an intensive regimen (3 h per day). The intensive regimen includes neuromotor physiotherapy sessions, 40 min each session, twice a day except for Saturdays when it is only one/day, 6 days a week. Rehabilitation treatments begin within 24 h after admission, and each participant is usually treated by the same therapists. Daily trainings targeting neuropsychological function, swallowing and bladder function are also part of the rehabilitation program. The part of TAU program focused on upper limb/hand motor re-learning will be monitored and possible variations (type of rehabilitation training) will be discussed with the clinicians who are in charge for participants rehabilitation program and are blinded to study intervention allocation of each participant.

We do not expect the BCI-MI and Control-MI interventions to cause adverse effects (non-invasive procedure; no drug-administration) and both intervention delivery will be under the care of specialized personnel (physiotherapists, neurophysiology technician experts in patient EEG recordings). Our previous experience with BCI training delivery in subacute stroke participants is encouraging since training was well-tolerated by the participants and no dropouts were reported [8, 12].

To monitor any possible deviation from the intervention training protocols (eg., missing sessions for intervening illness), a report form for each participants will be compiled at each planned training session by the training personnel and it will be part of the CRF (CRF- Outcome; details in Data collection and management section). During the follow-up period, clinicians (project staff) will monitor any home-based rehabilitative intervention (specific form in the CRF), clinical status (other disease/disorders/trauma) that can affect the upper limb motor function and possible admission to emergency room by means of regular phone-interviews.

### Statistical analysis

#### Sample size

The sample size was calculated on the basis of the primary hypothesis, that is experimental intervention (BCI-MI training) is superior in improving the primary outcome measure (UE-FMA at T1). Based on preliminary findings [8] in FMA scores in experimental vs control group (mean ± SD: 44 6 ± 34.7 vs 19.8 ± 19.8), alpha level at 5%, statistical power at 80%, and one-tailed t-test, 18 participants for group are needed. To account for possible loss at follow-up (assuming a drop-out rate = 30%), 24 participants per group will be enrolled in the study.

#### Primary analysis

Baseline characteristics will be described by summary statistics for each study group. The primary analysis will be in intention-to-treat (ITT) population on FMA primary outcome at T1. The ITT will include all randomized participants who will perform (minimum) 1 training session (both Experimental and Control intervention). T-test for independent groups will be used for comparisons and statistical significance will be assessed by one-tailed test (superiority).

#### Secondary analyses

Differences between experimental groups in FMA outcome will be assessed in the Per protocol (PP) population by including participants with an adherence to study protocols for intervention that is fixed at minimum 9 intervention sessions delivered within 6 weeks. In the secondary analyses, secondary outcomes will be compared between groups in ITT population at T1. T-test for independent groups will be used for continuous variables. Non-parametric tests will also be used. Differences among groups in categorical data will be assessed by chi-square test or Fisher’s exact probability test. To define whether and to what extent the efficacy of the BCI intervention on FMA score can be sustained long-term after the end of intervention–ie, in a 6-months follow-up, the analysis of variance (ANOVA) for repeated measures with group as between subject factor and time as within subject factor will be applied. In presence of missing at random data, mixed models will be used for statistical analyses. McNemar test will be used for analysis of repeated measures of categorical variables. Post-hoc comparisons will be performed.

The occurrence of MCID for FMA of 7 points from baseline/T0 to T1 (> 7 good recovery, < 7 bad recovery) [25] will be compared between groups by Fisher’s test.

To identify clinical and/or neurophysiological determinants of good recovery (improvement of 7 points in MCID) in participant response to a BCI-based treatment, a forward-stepwise binary logistic regression will be performed, and odds ratios and 95% confidence intervals will be calculated. A probability score will be defined to assess likelihood of success based on determinants of response. To reduce the dimensional space of the variables entered in the computation of outcome prediction, variable selection algorithms (e.g. Least Absolute Shrinkage and Selection Operator – LASSO regression) will be used to select the most informative features to be included in the predictive model. All analyses will be carried out also on PP population.

A blinded interim analysis for sample-size re-calculation (when first 20 participants who have completed FMA assessment at T1) will be performed to check congruity of data used in sample size calculation.

Further explorative analyses will be carried out on primary and secondary outcomes in subgroups of participants identified by strata used in randomization procedure. The statistical methods adopted for primary and secondary analyses will be used. To investigate possible effect of number/amount of training sessions received, comparisons among sub-groups of BCI participants defined according to number of training sessions [1–12] will be carried out on primary and secondary outcomes by using the same statistical methodologies adopted in primary and secondary analyses. All analyses will be under the expert supervision of the partner ISS and SAP.

### Data collection and management

An ad hoc CRF is implemented for all type and timing assessments. Specifically, the CRF will consist of two sections: i) the “Baseline and Randomization Section” which will contain each participant demographics and clinical data; all collected data as for the screening section including the Informed Consent, the randomization form (assigned randomized treatment) (for details see T(− 1) in Fig. 1 and Table 1). This part will be filled in by unblinded personnel (ie., recruiting neurologists); ii) the Outcomes Section - which will not include data on assigned experimental treatment – contains all the collected outcome data from T0 to T4 and during intervention training (see Table 1) will be filled by blinded personnel, namely the outcome assessors (details in T0 to T4 in Fig. 1 and Table 1).

A specific standard operating procedure including time schedule, and instruction for management and compilation of the CRF will be used. All study staff responsible for outcome assessment (neurologists, neuropsychologists, neurophysiologists, therapists, neuroimaging researchers and other researchers involved) and training (therapists and EEG technicians) will be trained on procedures to ensure validity and reliability of trial data collection. Adherence to time schedule and completeness of CRF will be monitored by the Clinical Trial Center (CTC) located at FSL. A clinical trial software for capturing CRFs, monitoring and managing clinical data and extracting data for analysis and reporting will be used.

### Confidentiality

Confidentiality and Privacy will be managed according to Italian National Law.

Personal data are regarded as strictly confidential. Original paper CRFs containing study data are stored at FSL and subjected to all security regulation and backup as clinical/medical records. The access to all study files is restricted to FSL staff involved in the study. Research staff at FSL enter data on a secure web-based electronic database which will be implemented by the ISS partner. Data are entered using participant-unique study codes only (anonymization). The blinded evaluators are prohibited from accessing any treatment related information in the database, including randomization assignment. Access to database is protected by password and limited to staff involved in the study at each research sites (FSL, ISS, SAP). The Promotoer system (with and without BCI modality) records EEG/EMG data from each participant and for each training session; data will be stored by unique study code only.

### Trial monitoring

Database collection within and between Experimental and Control intervention group will be monitored by the CTC that is independent from the study staff and sponsor. Trial responsible will be alerted if any deviation occurs. Any modifications to the protocol which may impact on the conduct of the study, including changes of study objectives, study design, participant population, sample sizes, study procedures, or significant administrative aspects will require a formal amendment to the protocol. Such amendment will be agreed upon by principal investigator and approved by the Ethics Committee prior to implementation and notified to the National Ministry of Health (sponsor).

### Dissemination of results

Main results will be subjected to publications on scientific peer-reviewed journals; results will be also presented at clinical neuroscience and/or neuroengineering (Society for Neuroscience conference; BCI international conference; IEEE; National Society for Neurorehabilitation) conferences. Media and public outreach are planned.

## Discussion

In addition to medical management, early stroke rehabilitation is initiated with the ultimate goal of achieving better recovery to reduce disability during the years that follow. Despite the recent advancement in rehabilitation medicine, the cost-efficiency of stroke rehabilitation care remains a crucial issue that burdens health care systems. The Promoter study aims at providing evidence for a BCI-assisted motor imagery training efficacy on the short and long-term post-stroke. Further aims are to seek for neurophysiological, neuroanatomical and clinical determinants of the outcomes of a BCI technology-based intervention that effectively boosts upper limb motor recovery in a short/long-term. Alongside with clinical efficacy, the knowledge about these intervention-related aspects is paramount to implement a future effective usage of the BCI technology in stroke motor rehabilitation management.

## Data Availability

Not Applicable.

## Abbreviations

ADM: Abductor Digiti Minimi
ARAT: Action Research Arm Test
BCI: Brain-Computer Interface
CMAP: Compound Muscle Action Potentials
CRF: Case Report Form
CTC: Clinical Trial Center
EEG: Electroencephalography
EMG: Electromyography
FMA: Fugl-Meyer Assessment
FSL: Fondazione Santa Lucia
ISS: Istituto Superiore Sanità
ITT: Intention-To-Treat
MAS: Modified Ashworth Scale
MCID: Minimal Clinically Important Difference
MEP: Motor Evoked Potential
MI: Motor Imagery
MMT: Manual Muscle Test
NIHSS: National Institute of Health Stroke Scale
NRS: Numeric Rating Scale
PDC: Partial Directed Coherence
PMCT: Peripheral motor conduction time
PP: Per Protocol
PSD: Power Spectral Density
QUEST: Quebec User Evaluation of Satisfaction with assistive Technology
RCT: Randomized Controlled Trial
SAP: Sapienza University of Rome
SD: Standard Deviation
SPIRIT: Standard Protocol Items: Recommendations for Interventional Trials
SUS: System Usability Score
TAU: Treatment As Usual
TMS: Transcranial Magnetic Stimulation
TT: Token Test
UE-FMA: Fugl-Meyer Assessment score for Upper Extremity
